# The relationship between well-being and digital health literacy in university students from Romania

**DOI:** 10.1093/eurpub/ckac129.711

**Published:** 2022-10-25

**Authors:** M Ungureanu, MA Coman

**Affiliations:** 1 Department of Public Health, Babes-Bolyai University Cluj-Napoca, Cluj-Napoca, Romania; 2 Center for Health Workforce Research & Policy, Babes-Bolyai University Cluj-Napoca, Cluj-Napoca, Romania

## Abstract

**Background:**

Digital Health Literacy (DHL) gained traction in recent years in the health promotion and well-being field as a possible protective factor.

**Methods:**

A cross-sectional survey was conducted using a self-reported web-based questionnaire on students enrolled at a university from Romania between December 2020 and February 2021. Descriptive statistics, correlation tests, and logistics regressions were employed to analyze the relationship between DHL, well-being, subjective social status (SSS), and future anxiety among students.

**Results:**

The data set included 1381 valid surveys (out of 1877 total surveys) completed by students aged between 18 and 39 years (mean = 21.9, SD = 3.701), the majority (69%) being males. Most of the students were studying at Bachelor level (83%), while the rest were Master or PhD students. Responses showed that 49% of the students expressed low well-being, 48% expressed a high level of future-anxiety and 59% considered they have a low SSS. For the DHL subscale of evaluating the reliability of the information, 56% of students had limited DHL, while for the subscale of determining the relevance of information, 64% of students presented limited DHL. The DHL subscale of adding self-generated content showed the highest correlation with well-being, followed by the determining relevance of the information subscale. Sufficient DHL was associated with higher levels of well-being when controlling for age, gender, and study program.

**Conclusions:**

Well-being is influenced by actions such as adding self-generated health content, determining the relevance of health information, and anxiety for the future, all being important actions in health promotion. Individual factors such as age and gender are also relevant in mediating the relationship between DHL and well-being.

